# The Role of Adjuvant Therapies in Improving Outcomes for pT1-3N0 Oral Squamous Cell Carcinoma With Tumour-Free Margins and Perineural Invasion

**DOI:** 10.7759/cureus.76071

**Published:** 2024-12-20

**Authors:** Gauri Gupta, Ashish Sharma, Himanshu Bhutani

**Affiliations:** 1 Oral and Maxillofacial Surgery, ITS Dental College, Hospital and Research Centre, Greater Noida, IND

**Keywords:** cisplatin, concomitant chemoradiation therapy, neck dissection, oral squamous cell carcinoma, perineural spread

## Abstract

Introduction

The role of concomitant chemoradiation therapy (CTRT) or radiation therapy (RT) is not well defined in operated cases of oral squamous cell carcinoma (OSCC) with positive perineural spread. The purpose of the study was to determine whether the use of concurrent CTRT or RT would enhance the five-year disease-free survival of patients with positive perineural invasion (PNI).

Materials and methods

Data were analysed retrospectively from January 2014 to December 2023. Patients were placed into three groups: surgery only, surgery with RT, and surgery with concomitant CTRT. In all, 180 cases of pT1-3N0 and pT1-3N+ OSCC patients had tumour-free margins, of which 24 cases (13.4%) had perineural invasion. Based on treatment modalities, 45.8% of the cases underwent surgery with CTRT (group III), 33.3% opted for surgery with RT (group II), and 20.9% underwent surgery only (group I). Five-year recurrence-free survival was analysed among the three groups using the Kaplan-Meier model.

Results

There was no significant difference among the three groups in terms of recurrence (p = 0.817) or five-year survival rate (p = 0.0935).

Conclusion

Altogether, the data seem to indicate that radical surgical resection alone should be considered sufficient treatment for OSCC patients with pT1-3N0 disease, even in the presence of perineural invasion. Thus, it can be concluded that the addition of concomitant CTRT or RT does not significantly increase the five-year disease-free survival of patients with OSCC with positive PNI.

## Introduction

Positive perineural invasion (PNI) is a distinct entity where malignancy spreads due to its close proximity to the nerves [1-4]. This entity is considered an independent prognostic factor for local and systemic spread without the involvement of lymph nodes or perivascular spread [5-8]. The spread may involve the entire length of the nerve tissue, obstructing the management of the malignancies and significantly reducing the survival rate [9]. To date, no set protocols have been established for patients with oral squamous cell carcinoma (OSCC) with perineural spread. Further controversies exist in the management of patients with OSCC pT1-3N0 or pT1-3N+ having clear margins and perineural spread [10]. Some authors advocate the role of adjunct therapies, mainly radiation and chemotherapy, along with surgery in managing the perineural spread, whereas others consider surgical management the most appropriate treatment for managing the perineural spread [11-13].

Keeping in mind the existing disagreements, a retrospective study was planned from January 2014 to December 2023 on OSCC pT1-3N0 and pT1-3N+ patients with negative margins and perineural spread. The prognosis, recurrence rate, and five-year survival of patients were analysed using various treatment modalities [14]. The treatment modalities compared were surgery, surgery with radiotherapy (RT), and surgery with concurrent chemoradiation therapy (CTRT). A null hypothesis was proposed, advocating that no significant difference exists in terms of recurrence and survival rate among the various treatment modalities in patients with OSCC with perineural spread.

## Materials and methods

Study design and setting

A retrospective analysis was conducted from January 2014 to December 2023 on OSCC patients who had undergone surgery and were diagnosed as positive for perineural invasion at a single centre. The research was authorised by the ethical committee and adhered to the guidelines of the Helsinki Declaration.

The medical records of all patients with oral cavity cancer were examined. American Joint Committee on Cancer staging protocols were used to classify the patients. Patients older than 18 years, with positive perineural invasion, clear margins, and tumour sizes T1-T3 with N0-N+ necks, were included in the study. However, patients with T4-size tumours, perivascular invasion, or extracapsular spread were excluded from the study. After meeting the inclusion and exclusion criteria, patients were divided into three groups: in group 1, patients underwent surgery only and deferred any other form of treatment; in group 2, patients underwent combined surgery and radiation therapy but deferred chemotherapy; whereas in group 3, patients underwent combined surgery with concurrent chemotherapy (single-drug Cisplatin) and radiotherapy.

Surgical protocol 

The surgical methods performed in the study included primary tumour excision with clear margins, supraomohyoid neck dissection (SOND) for N0 necks, and modified neck dissection for N+ necks. However, the bilateral neck was addressed if lesions crossed the midline. Adjuvant treatment modalities in the form of postoperative radiotherapy or concurrent chemoradiotherapy were considered for all patients with positive perineural invasion, as per the guidelines of the institutional tumour board. These adjuvant treatment modalities were not mandatory but were left to the patients to decide based on their medical and financial status.

The final decision regarding adjuvant therapies was made three to four weeks after the surgery. Radiotherapy targeted the entire tumour bed area with 2 cm margins and the regional lymphatics. A total dose of 60 gray was administered, with 2 gray per fraction each day for five days a week. Cisplatin, at 100 mg per kg of body weight, was used for concomitant chemotherapy. The concomitant chemotherapy was given on days 1, 20, and 40, alongside the radiotherapy regimen. All identified patients were followed up for a duration of five years or until death. Recurrence and survival were compared amongst the various groups.

Statistical analysis

A null hypothesis was proposed stating that no difference exists among the three groups in terms of recurrence rate and five-year survival rate. An alternate hypothesis was put forward, stating that a significant difference does exist among the groups.

If p > 0.005, the null hypothesis was accepted; otherwise, the alternate hypothesis was considered. Statistical data were analysed using IBM SPSS Statistics for Windows, Version 23 (Released 2015; IBM Corp., Armonk, New York). The statistical tests used for analysing data included mean, median, standard deviation, and the chi-square test. The Kaplan-Meier model was used for recurrence and survival analysis. The log-rank test evaluated the equality of recurrence and survival by weighing all time parameters; Breslow tests measured recurrence and survivability by counting the number of patients at risk; and the Tarone-Ware test was used for recurrence and survival by weighing all time parameters by the square root of the number of patients at risk.

## Results

In all, 180 cases of pT1-3N0 and pT1-3N OSCC patients had tumour-free margins, of which 24 cases (13.4%) exhibited perineural invasion. Based on treatment modalities, 11 (45.8%) underwent surgery with CTRT (group III), 8 (33.3%) opted for surgery with radiotherapy (group II), and 5 (20.8%) underwent surgery only (group I). Age-wise distribution of groups revealed a mean age of 47 ± 9.69 years for group I patients, 55.0 ± 10.23 years for group II patients, and 55.63 ± 8.5 years for group III patients. In terms of gender, men were the dominant population in all three groups, with 4 (80%) males in group I, 5 (62.5%) in group II, and 8 (72.7%) in group III (Tables 1, 2).

**Table 1:** Distribution according to positive perineural invasion (PNI)

Distribution	N	%
PNI-positive	24	13.4
PNI-negative	15.6	86.6

**Table 2:** Distribution according to age and gender PNI: perineural invasion, RT: radiotherapy, CT: chemotherapy, SD: standard deviation.

	Gender				Age		PNI-Positive Patients	
	Male		Female					
Treatment Modalities	N	%	N	%	Mean	SD	N	%
Surgery	4	80	1	20	47	9.69	5	20.8
Surgery+RT	5	62.5	3	37.5	55.0	10.23	8	33.3
Surgery+RT+CT	8	72.7	3	27.3	55.63	8.51	11	45.8

All patients in each group underwent neck dissections, either in the form of SOND (2 cases, 40%) or modified neck dissection (4 cases, 60%). The average depths of the tumours in the three groups were 9.2 ± 3.38 mm for group I, 9.12 ± 9.1 mm for group II, and 9.36 ± 3.10 mm for group III.

**Figure 1:**
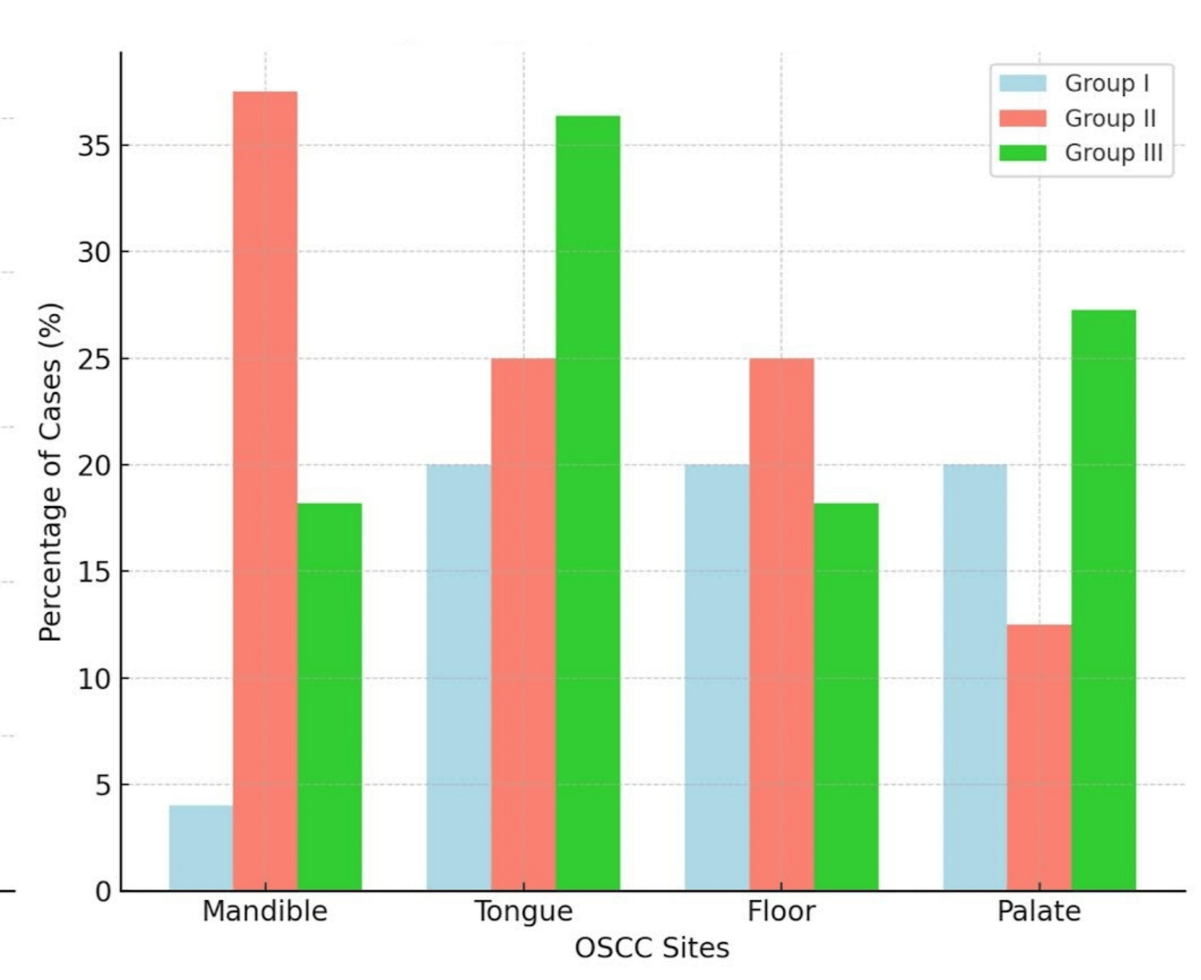
Distribution according to site OSCC: oral squamous cell carcinoma.

Based on tumour size, the mean size for group I was 3.46 ± 1.47 cm, for group II was 3.16 ± 1 cm, and for group III was 3.88 ± 1.29 cm. Analysing the sites affected by OSCC showed that in group I, 2 (40%) cases involved the mandible, 1 (20%) the tongue, 1 (20%) the floor, and 1 (20%) the palate. In group II, 3 (37.5%) patients had mandibular OSCC, 2 (25%) had tongue OSCC, 1 (12.5%) had palatal OSCC, and 2 (25%) had lesions on the floor of the mouth. Similarly, 2 (18.18%) of group III patients had mandibular OSCC, 4 (36.36%) had tongue OSCC, 2 (18.18%) had floor lesions, and 3 (27.27%) had the palate involved (Table 3, Figure 1).

**Table 3:** Distribution according to site, depth and size RT: radiotherapy, CT: chemotherapy, SD: standard deviation.

	Site								Tumour Depth (mm)		Tumour Size (cm)	
	Mandible		Tongue		Palate		Floor of the Mouth					
	N	%	N	%	N	%	N	%	Mean	SD	Mean	SD
Surgery	2	40	1	20	1	20	1	20	9.2	2.38	3.64	1.47
Surgery+RT	3	37.5	2	25	1	12.5	2	25	9.12	3.35	3.16	1.00
Surgery+RT+CT	2	18.18	4	36.36	3	27.27	2	18.18	9.36	3.10	3.88	1.29

Recurrence and survival analysis

Analysis for Recurrence

**Figure 2:**
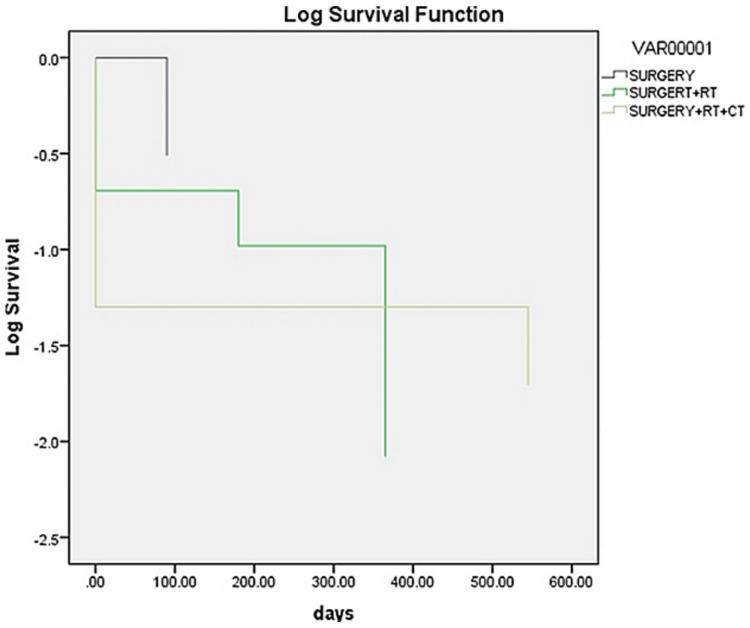
Comparison of recurrence rate among the three treatment groups RT: radiotherapy, CT: chemotherapy.

Log-rank, Breslow, and Tarone-Ware tests were conducted to determine if there were differences in recurrence among the three different treatment modalities. The significant values of the tests applied were p = 0.876 (Log-rank test), p = 0.196 (Breslow test), and p = 0.513 (Tarone-Ware test). Since p > 0.05, there was no statistical significance (Figure 2).

Analysis for Survival

**Figure 3:**
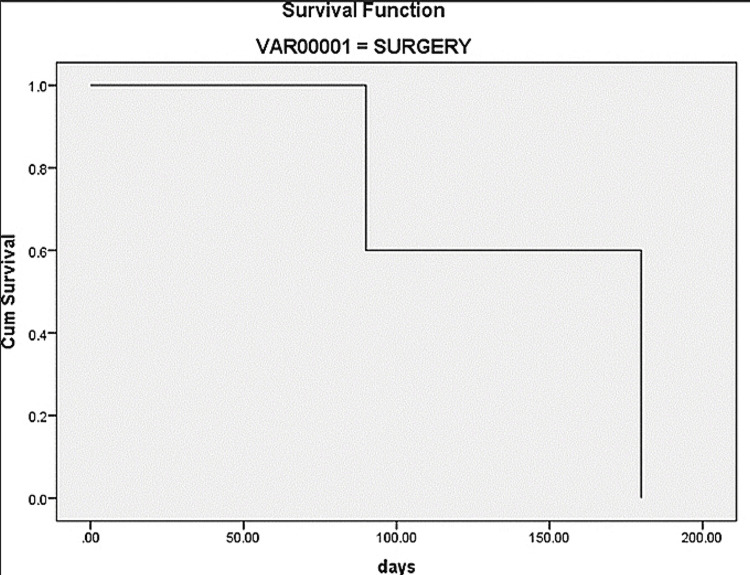
Survival rate in surgery-only group Cum: cumulative.

**Figure 4:**
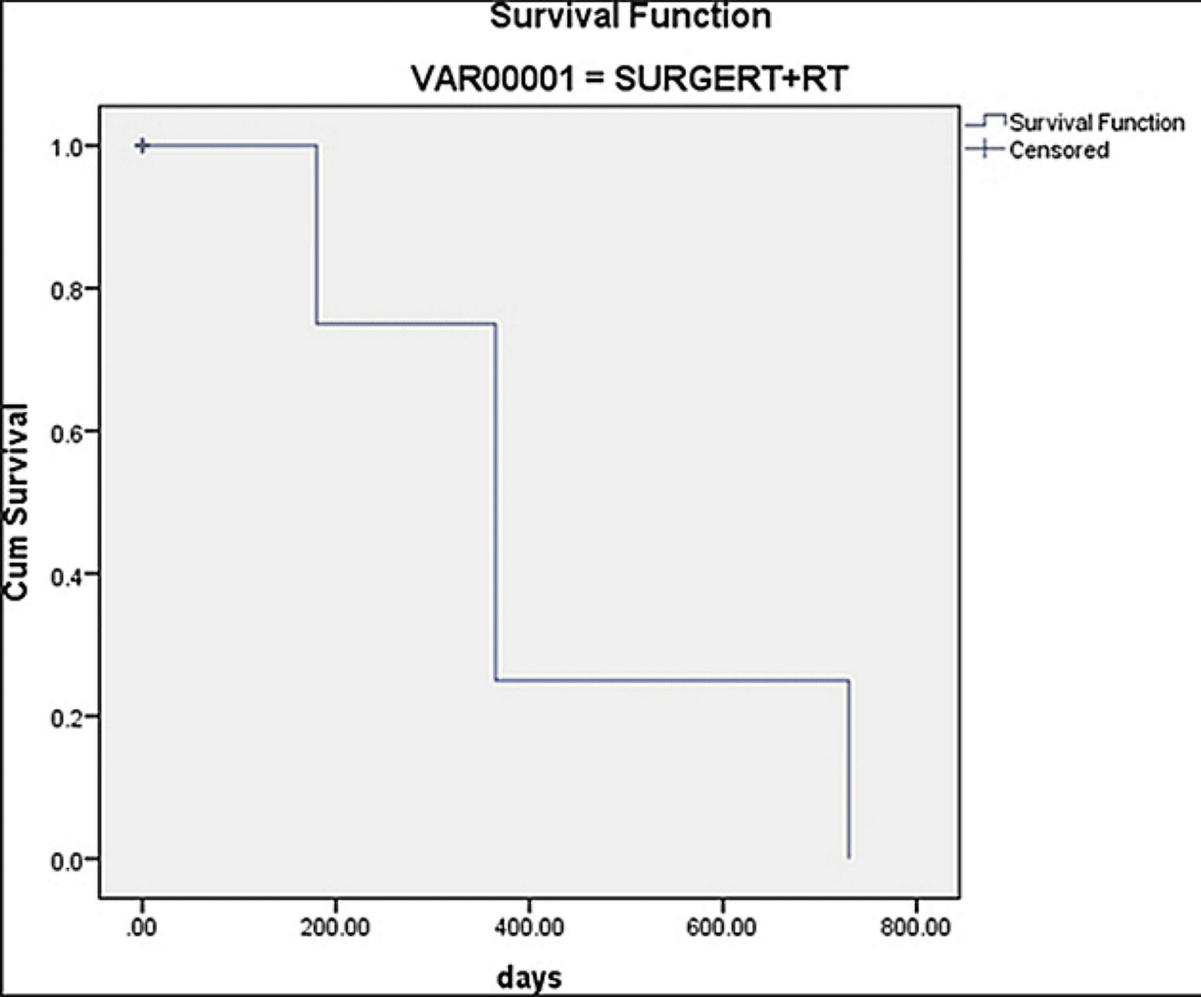
Survival rate in surgery and radiation therapy groups Cum: cumulative.

**Figure 5:**
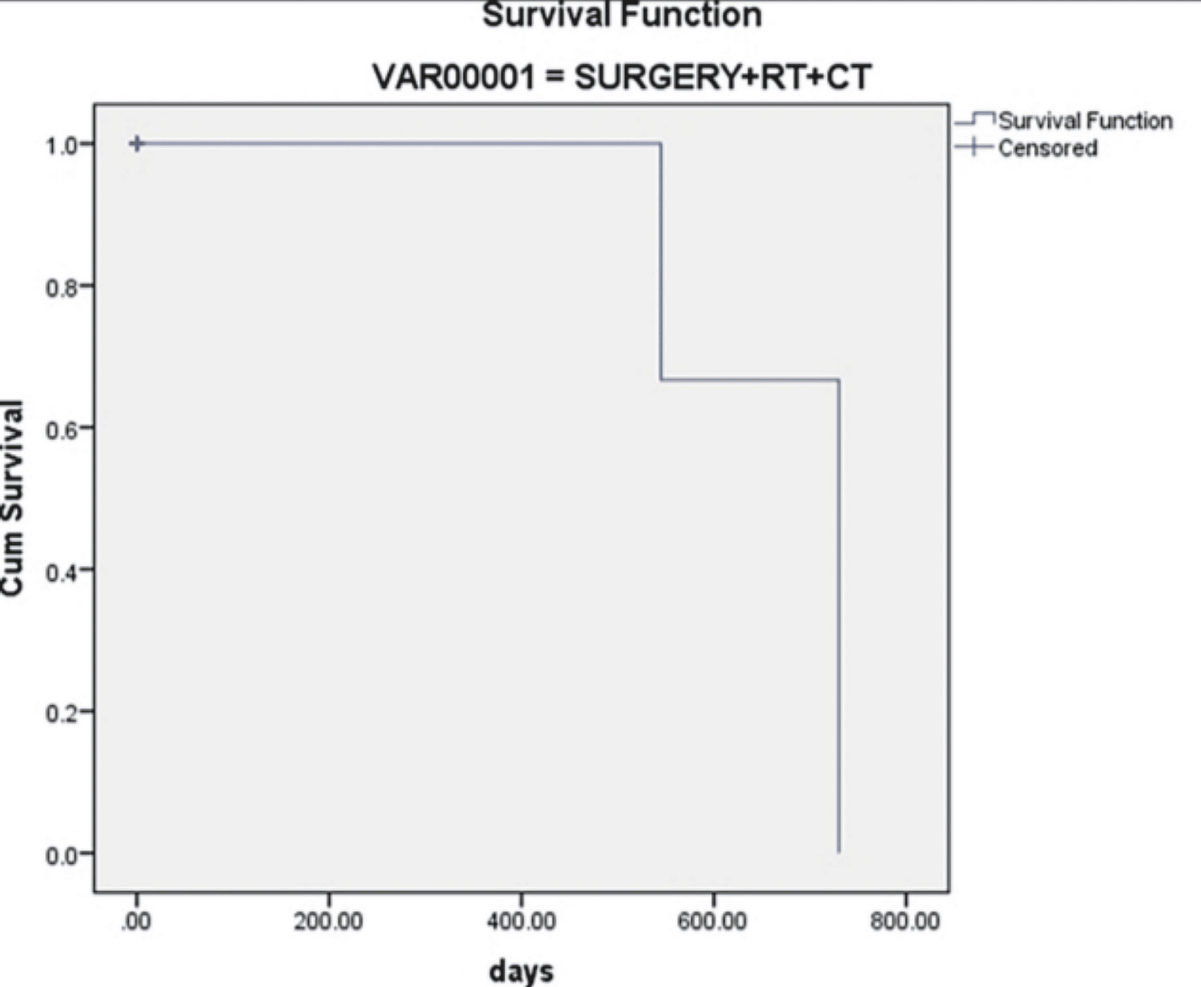
Survival rate in surgery, radiation, and chemotherapy groups Cum: cumulative.

Log-rank, Breslow, and Tarone-Ware tests were conducted to determine the differences in survival among the three treatment groups. The significant values of the tests applied were p = 0.91 (Log-rank test), p = 0.166 (Breslow test), and p = 0.713 (Tarone-Ware test). Because the significant values of the tests were greater than 0.05, the statistical difference failed to reach the level of significance between the treatment groups (Figures 3-5).

Because p > 0.05, there was no significant difference, and the null hypothesis was accepted. Additionally, the alternate hypothesis, which stated that significant differences exist in terms of recurrence and five-year survival, was rejected.

## Discussion

The role of perineural spread is considered an independent prognostic marker for OSCC patients, necessitating a definitive set of protocols for treatment. This type of tumour spread is characterised by aggressive behaviour and recurrence. Considering these issues, the present study was conducted to compare prognosis, recurrence rate, and five-year survival rate for patients with perineural invasion in OSCC treated with different treatment modalities. A null hypothesis was proposed, advocating that no significant difference exists in terms of recurrence rate and five-year survival rate amongst the various treatment modality groups.

The results of the prospective study confirm that perineural spread is an independent marker for poor prognosis. Woolgar described that OSCC with PNI in minor nerves (<1 mm) or major nerves reduces both the survival rate and recurrence [15-17].

Because p > 0.05, no significant difference exists amongst the treatment groups in terms of recurrence and five-year survival rate, so the null hypothesis was accepted. A study conducted by Liao et al. [9] revealed no significant difference in the five-year control (p = 0.1936) and survival (p = 0.5580) rates between OSCC patients with perineural invasion and those without it. The inclusion of adjuvant radiation had no effect on the five-year localised control rate (p = 0.3170) or total survival rate (p = 0.0935) in individuals with perineural invasion.

Similar to the present study, Liao et al. concluded that the addition of adjuvant radiotherapy did not significantly alter the five-year local control rate or the overall survival rate.

Laske et al. [12] compared five-year survival and recurrence rates in a perineural invasion group and a control group. The treatment modalities used were surgery, surgery with RT, and surgery with concomitant CTRT. Postoperative radiotherapy doses ranged from 60 gray to 66 gray, applied in 30 to 33 fractions, five times a week, and administered to the tumour bed area. Elective treatment of the nodal pathways included doses of 54 gray (low risk) or 60 gray (high risk). Cisplatin was utilised as the concomitant chemotherapy in all patients, except for two cases in the PNI group, where Cetuximab was employed. The results concluded that significant differences exist in the recurrence-free survival rate between the two groups (i.e., the PNI-free and PNI-positive groups). The PNI-free group had a higher survival rate and a longer recurrence-free period. This study concluded that additional therapies may not increase survival in PNI-positive groups. The results of both studies align with the present study and support the fact that adjunct therapies do not significantly improve the five-year recurrence-free survival of patients with PNI [18,19].

Although a wide range of treatment options exist for managing PNI in OSCC, no specific guidelines have been established for PNI management. Radiation therapy may fail because it does not target or insufficiently targets the nerves with PNI. Chemotherapy may fail in PNI because vascularisation may be insufficient to target the cancer cells. Overall, the findings of the research support the notion that radical resection alone, especially in the presence of perineural invasion, should be regarded as adequate therapy for OSCC patients with pT1-3N0 and pT1-3N+ disease, as recommended by Liao et al. The role of primary nerve resection in controlling PNI needs to be further investigated [20,21].

Limitations of the study

The criteria for the administration of adjuvant therapies in OSCC remain controversial. Studies with larger sample sizes are required to analyse the efficacy of adjuvant therapies in OSCC with perineural invasion. However, it remains to be established whether OSCC patients with perineural invasion alone may have more favourable tumour biology. In this regard, a prospective study is warranted to address some unresolved issues.

## Conclusions

The study concludes that concomitant CTRT or RT does not significantly increase the five-year disease-free survival of OSCC patients with positive PNI. PNI is a widely accepted clinical and histopathological feature associated with aggressive disease and poor prognosis.

Although it represents a distinct third mode of tumour metastasis, alongside lymphatic and blood vessel invasion, PNI is not well studied. A lack of experimental models or even an accurate definition of PNI has hindered progress in understanding the mechanisms of this phenomenon. Specifically, the question remains unresolved as to whether perineural invasion alone may be an independent prognosticator in OSCC patients with pT1-3N0. In this context, the use of adjuvant therapies must be readdressed.
